# Impact of an immersive, interactive medical education initiative on guideline-based retinal disease management knowledge/competence and effectual practice change

**DOI:** 10.1186/s12886-023-03034-9

**Published:** 2023-06-22

**Authors:** Rishi P. Singh, Lauren Welch, Nicole L. Longo, Matt Frese

**Affiliations:** 1 Cleveland Clinic Lerner College of Medicine, Cleveland, OH USA; 2 Cleveland Clinic Martin Health, Stuart, FL USA; 3 Med Learning Group, 17th St #4, New York, NY 10011 USA

**Keywords:** Retinal disease, Anti-vascular endothelial growth factor, Continuing education, Diabetic retinopathy, Diabetic macular edema, Age-related macular degeneration, Macular degeneration, Retinal vein occlusion, Guideline-based treatment, Anti-VEGF

## Abstract

**Background:**

Retinal diseases, including wet or dry age-related macular degeneration, diabetic macular edema, and diabetic retinopathy (DR), are underdiagnosed and undertreated in the United States. Clinical trials support the effectiveness of anti-vascular endothelial growth factor (anti-VEGF) therapies for several retinal conditions, but real-world data suggest underuse by clinicians, resulting in patients experiencing poorer visual outcomes over time. Continuing education (CE) has demonstrated effectiveness at changing practice behaviors, but more research is needed to understand whether CE can help address diagnostic and treatment gaps.

**Methods:**

This test and control matched pair analysis examined pre-/post-test knowledge of retinal diseases and guideline-based screening and intervention among 10,786 healthcare practitioners (i.e., retina specialists, ophthalmologists, optometrists, primary care providers, diabetes educators, pharmacists/managed care specialists, and other healthcare providers, such as registered nurses, nurse practitioners, and physician assistants) who participated in a modular, interactive CE initiative. An additional medical claims analysis provided data on practice change, evaluating use of VEGF-A inhibitors among retina specialist and ophthalmologist learners (n = 7,827) pre-/post-education, compared to a matched control group of non-learners. Outcomes were pre-/post-test change in knowledge/competence and clinical change in application of anti-VEGF therapy, as identified by the medical claims analysis.

**Results:**

Learners significantly improved knowledge/competence scores on early identification and treatment, identifying patients who could benefit from anti-VEGF agents, using guideline-recommended care, recognizing the importance of screening and referral, and recognizing the importance of early detection and care for DR (all *P*-values = 0.003 to 0.004). Compared with matched controls, learners’ incremental total injections for anti-VEGF agents for retinal conditions increased more after the CE intervention (*P* < 0.001); specifically, there were 18,513 more (new) anti-VEGF injections prescribed versus non-learners (*P* < 0.001).

**Conclusions:**

This modular, interactive, immersive CE initiative resulted in significant knowledge/competence gains among retinal disease care providers and changes in practice-related treatment behaviors (i.e., appropriate consideration and greater incorporation of guideline-recommended anti-VEGF therapies) among participating ophthalmologists and retina specialists compared to matched controls. Future studies will utilize medical claims data to show longitudinal impact of this CE initiative on treatment behavior among specialists and impact on diagnosis and referral rates among optometrists and primary care providers who participate in future programming.

## Background

Due in part to the growth of the aging population, retinal diseases are becoming increasingly common in the United States, particularly wet or dry age-related macular degeneration (AMD), diabetic macular edema (DME), diabetic retinopathy (DR), and branch or central retinal vein occlusion (BRVO; CRVO) [1]. A retrospective study of more than 3 million eyes found prevalence rates of approximately 16% for dry AMD, 9.5% for wet AMD, 9% for DME, 8% for DR without DME, and 2% for BRVO and CRVO each [1]. The number of U.S. adults experiencing vision impairment or blindness has been projected to double by 2050 [2]. Retinal diseases represent a significant burden to patients, leading to increased ocular morbidity, vision deficits, and potentially permanent blindness. Not surprisingly, patients with vision impairments often experience reduced vision-related quality of life (QOL), impairments in social relationships, and significant limitations in activities of daily living, such as reading, driving, and using the computer, which in turn can negatively affect their mood, functioning, and overall well-being [3–6,].

Compared with pivotal clinical trials, a well-recognized pattern of underutilization of screening procedures for retinal diseases has emerged in real-world settings, resulting in patients acquiring less vision gain [7–9]. There are also established patterns of underdiagnosis among distinct retinal disorders, including DR and AMD. For instance, DR is often not detected and diagnosed until after severe damage has occurred [8, 9]. In a sample of 1,288 eyes from 644 participants in a primary eye care ophthalmology or optometry clinic [7], the prevalence of undiagnosed AMD was 24.8%. Efforts to improve clinician screening and diagnosis of retinal disorders could reduce diagnostic delay and potentially result in faster and more appropriate treatment for patients with retinal diseases, giving them more opportunities to preserve functioning and QOL.

Even when screened for and diagnosed appropriately with retinal diseases, real-world patients may receive suboptimal treatments. This is especially true for anti-vascular endothelial growth factor (VEGF) therapies, which are guideline recommended for the treatment of AMD [10], DME [11], DR [12], and RVO [13]. The advent of anti-VEGF therapies (i.e., bevacizumab, ranibizumab, aflibercept, and brolucizumab) helped revolutionize the treatment landscape for AMD, including neovascular AMD (nAMD), by providing patients with a safe and efficacious therapy that results in significant improvements in vision and reduced vision loss [14]. Further, the use of laser treatment for conditions such as DR, DME, and RVO should be individualized in light of possible long-term treatment-related adverse effects, such as pain, rebound edema, worsening of disease, visual field loss, reduction in color vision and/or night vision, choroidal complications, and vision acuity that either declines or fails to improve [15–17]. This underscores the importance of offering anti-VEGF treatments as a therapeutic option with less potentially negative impact to long-term vision. Following the success of these therapies for the treatment of AMD, anti-VEGF drugs subsequently gained U.S. Food & Drug Administration approval for the treatment of DME, RVO, and DR [18].

Pivotal and other randomized controlled trials have consistently shown that timely dosing and consistent monitoring is critical for patients to optimally benefit from anti-VEGF injections [19–21]. Unfortunately, ample data from real-world studies suggest that outside of clinical trials, patients do not receive sufficient treatment and monitoring and, as a result, demonstrate significantly worse vision outcomes (e.g., fewer Early Treatment of Diabetic Retinopathy [ETDR] letter gains) than do patients in clinical trials [19, 20, 22–25]. One retrospective analysis of clinical practice patterns found more than 19,000 newly diagnosed patients with nAMD received a mean of 4.6, 5.1, and 5.5 annual injections for bevacizumab and a mean of 6.1, 6.6, and 6.9 annual injections for ranibizumab from 2008 to 2010 [19]. Furthermore, only 12% of patients receiving bevacizumab and 21% of patients receiving ranibizumab had at least 10 injections within a 12-month period [19]. In clinical trials, the mean rate of injection over the same period is much higher; for instance, the MARINA and ANCHOR clinical trials reported 12.3 injections and 11.2 injections, respectively, over the course of 12 months [19]. As a result of these discrepancies, short- and long-term vision acuity gains in real-world studies tend to be smaller than in clinical trials. In the MARINA trial, the average vision acuity gain at 2 years among patients in the treatment arm was 7 letters, and in the ANCHOR study, treatment patients gained at least 15 letters at 1 year [26]. By comparison, the multicenter UK Age-Related Macular Degeneration EMR Users Group real-world study of 12,951 eyes showed 2 letters gained, 1 letter gained, and 2 letters lost at 1-year, 2-year, and 3-year assessments, respectively [27].

These practice gaps (i.e., underscreening, underdiagnosis, and undertreatment with anti-VEGF therapy) underscore the importance of ongoing medical education to improve advanced outcomes in both knowledge/competence and practice behaviors among healthcare providers caring for patients with eye disease and in particular potentially vision-threatening retinal conditions. Medical education is a reasonable pathway toward inducing small to moderate degrees of practice change among healthcare providers, including eye care providers [28, 29]. One quality improvement education initiative for optometrists designed to enhance clinician knowledge, assessment, and evaluation and referral patterns for diabetic patients [30] was associated with significant increases in screening (i.e., dilated fundus examination) and significant decreases in improper follow-up instructions for diabetic retinopathy. More data are needed on continuing education (CE) programming specific to retinal diseases and its impact on patient care outcomes. However, research on CE in general has shown it to be effective in helping clinicians acquire and retain new knowledge, attitudes, skills, and behaviors, including improvements in prescribing behaviors, screening, counseling patients about lifestyle behaviors (e.g., smoking cessation, diet) and providing guideline-concordant care [29]. CE programs likely to have the greatest impact on healthcare provider performance are those that use multimedia, are interactive (e.g., audit and feedback), use live media rather than print, offer simulation, feature multiple exposure to content rather than single exposure, and use multiple education techniques rather than a single approach [29].

Although advanced outcomes at the performance or patient health level are invaluable in how they demonstrate the impact of CE, it can be challenging to connect the results from an individual education program with the overall learning continuum for a particular therapeutic area. However, when applied to learners from a variety of programming within a single therapeutic area, performance-level outcomes can more easily be embodied within overall strategic planning year on year. Medical claims analysis is one approach to analyzing performance-level outcomes across multiple educational activities of differing modalities; this could help demonstrate the impact of education on clinical practice and improve our ability to identify areas for continued focus. A claims-linked analysis of CE outcomes would be a novel contribution to the retinal diseases and ophthalmology fields in that: (1) it would add to the dearth of literature on CE programming and patient care outcomes in these disease states, and (2) it could help better quantify patterns of practice behavior change resulting from CE learning among multiple retinal disease and ophthalmology disciplines.

In 2019, Med Learning Group (MLG) launched VISION RELIEF, with independent educational grant support from Regeneron Pharmaceuticals, Inc., to provide an immersive, interactive education platform and resource repository for vision care specialists as well as non-specialist healthcare providers who encounter patients with retinal disease (e.g., primary care providers, diabetes care providers). MLG conducted 9 live/livestream and 10 online CE programs in 2021 alone as part of its multiyear VISION RELIEF educational initiative. To help address practice and knowledge gaps about retinal diseases and to better explore the impact of CE on knowledge and performance outcomes among eye care providers and educators, this test and control matched pair analysis examined change in knowledge/competence and practice behavior change, operationalized by change in guideline-recommended use of anti-VEGF agents, among VISION RELIEF learners over a 17-month span from 2020 to 2021.

## Methods

In 2021, there were 38,844 total learners across a variety of professions who accessed VISION RELIEF through 9 national conferences and via 14 enduring programs. For the knowledge/competency change analysis, 10,786 VISION RELIEF learners in each activity completed pre-/post-tests utilizing knowledge- and case-based questions, with access to the VISION RELIEF website containing supplementary educational tools. This included 3,285 learners representing retina specialists and ophthalmologists nationwide and 7,501 learners representing optometry, primary care diabetes educators, pharmacists/managed care, and other HCPs. All learners accessed VISION RELIEF from August 2020 to December 2021.

For the practice change analysis, 7,827 retina specialists and ophthalmologist learners participating in VISION RELIEF were matched to non-learner controls from an IQVIA database. Pairs were matched on geography, specialty, new injections with VEGF inhibitors, overall treating decile (e.g., how many times a learner has prescribed a therapy for a retinal disease), and anti-VEGF agent treating decile (e.g., how many times a learner has prescribed an anti-VEGF agent). The control group was derived from MLG providing a list of all learners to IQVIA. The study is based on the learners IQVIA finds in their database. The control group is then created from healthcare providers in their database who are matched to learners on the following criteria: Anti VEGF(A) inhibitors new prescriptions and new administrations volume and share during the pretest period; market new prescriptions and new administrations volume during the pretest period; healthcare provider specialty; healthcare provider geography (i.e., urban, suburban, or rural); active writing rate; anti VEGF(A) inhibitors decile groups (i.e., 0, 1–3, 4–6, 7–10); DR/AMD/RVO decile groups (i.e., 0, 1–3, 4–6, 7–10); and IQVIA diagnosis tier status. The analysis of practice change is based on learners who took part in VISION RELIEF between August 2020 and December 2021. To conduct this analysis, IQVIA analyzed treatment behavior by class of therapy 6 months prior to the learner entering the education intervention. If the learner participates in multiple VISION RELIEF programs, the timing is based on the first one they participated in. The participant’s behavior postparticipation in the activity until December 2021 was analyzed.

All methods were carried out in accordance with relevant guidelines and regulations. No incentives were provided to participants.

### Statistical approach

Univariate analyses were employed to compare pre- and post-test results on knowledge/competence among VISION RELIEF learners. Results only include data from learners who completed both pre and post data; thus, learners with missing data were excluded. Analysis of covariance, an F test from which IQIVA derived the confidence and p-values, was conducted to assess change in anti-VEGF injections between learners and matched controls/non-learners. The Bonferroni adjustment was not applied because the analysis is performed on the overall result, not on individual learners. The subgroup results are based on this overall, significant result and are not independent tests.

### Outcomes

The primary outcome was pre- and post-test change in knowledge/competence of retinal diseases DR/DME, AMD, and RVO and the application of guideline-based treatment recommendations for each. Additional outcomes focused on real-world clinical application of guideline-based treatment post education, analyzing change in practice behavior (i.e., application of anti-VEGF therapy). Outcomes in knowledge/competence change were examined by profession (e.g., optometry, ophthalmology, retina specialty care, primary care physicians, diabetes educators). Comparison of raw medical claims data among learners versus matched controls was used to identify the change in application of recommended anti-VEGF-A inhibitors among retina specialists and ophthalmologists.

### Intervention and testing

**Fig. 1:**
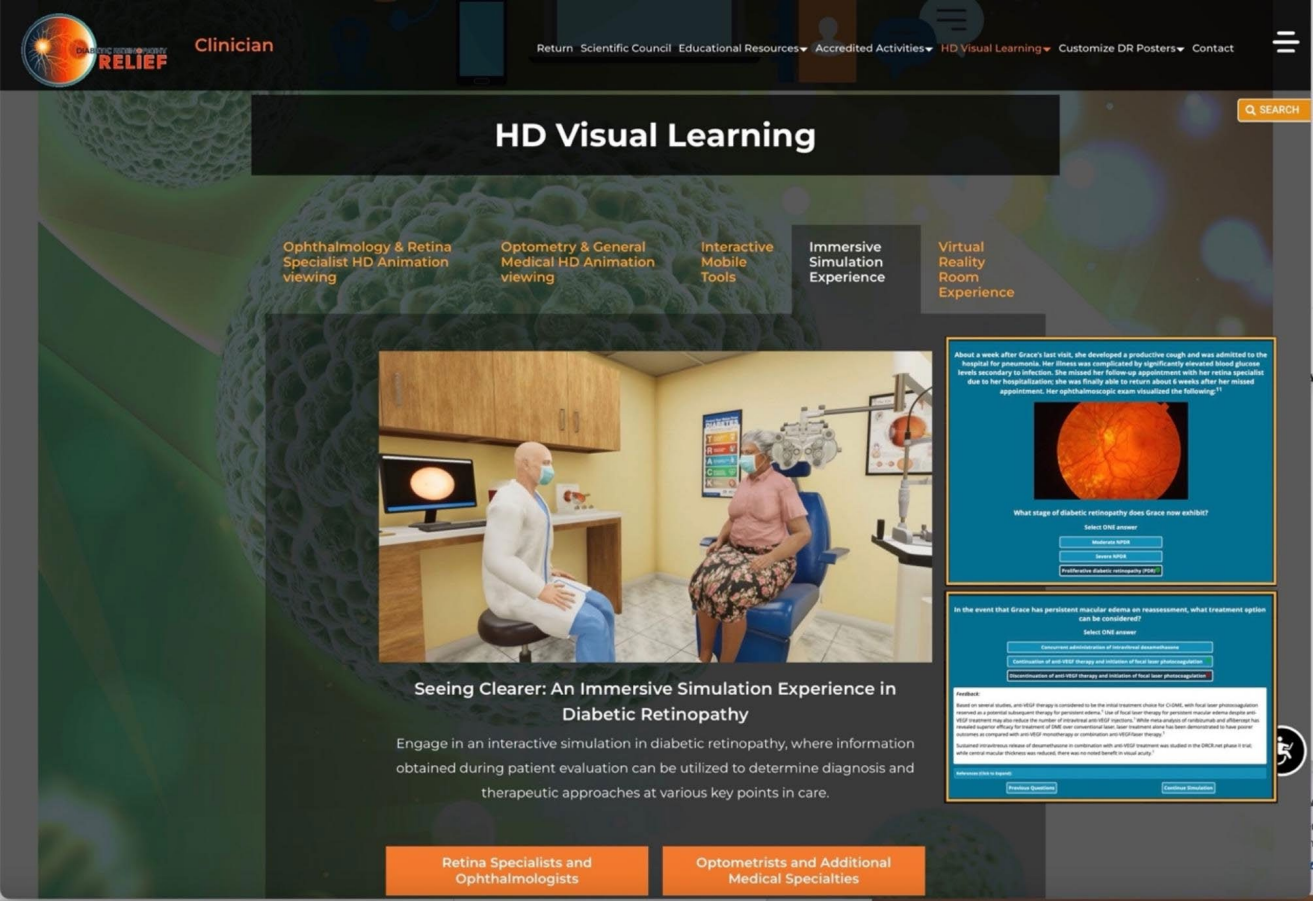
An Immersive Simulation for Improving Diagnostic Evaluation in Diabetic Retinopathy Images provided with permission by MLG, creators and curators of all VISION RELIEF content

**Fig. 2:**
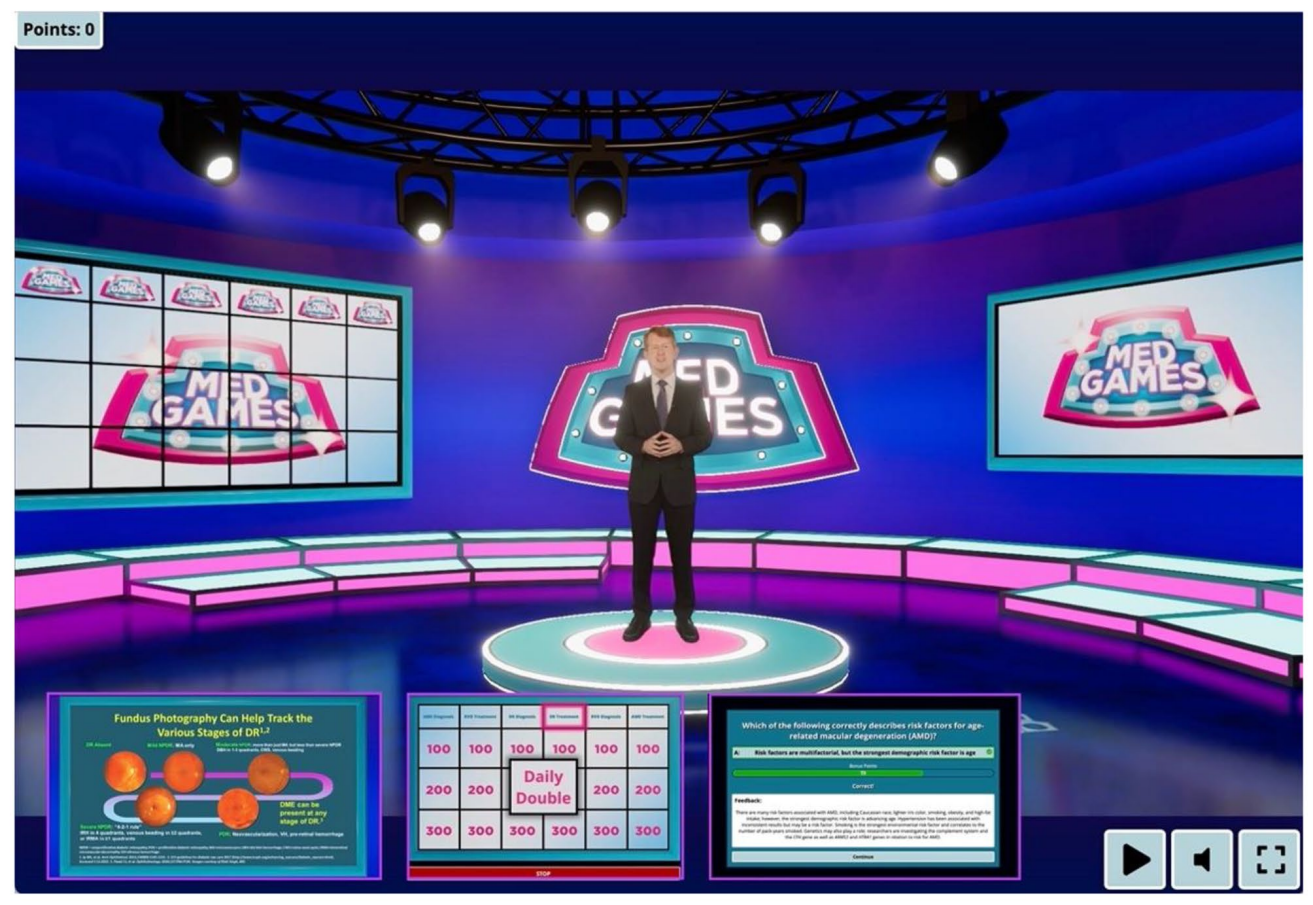
Med Games Interactive Module to Enhance Diagnosis and Treatment of Retinal Diseases Images provided with permission by MLG, creators and curators of all VISION RELIEF content

**Fig. 3:**
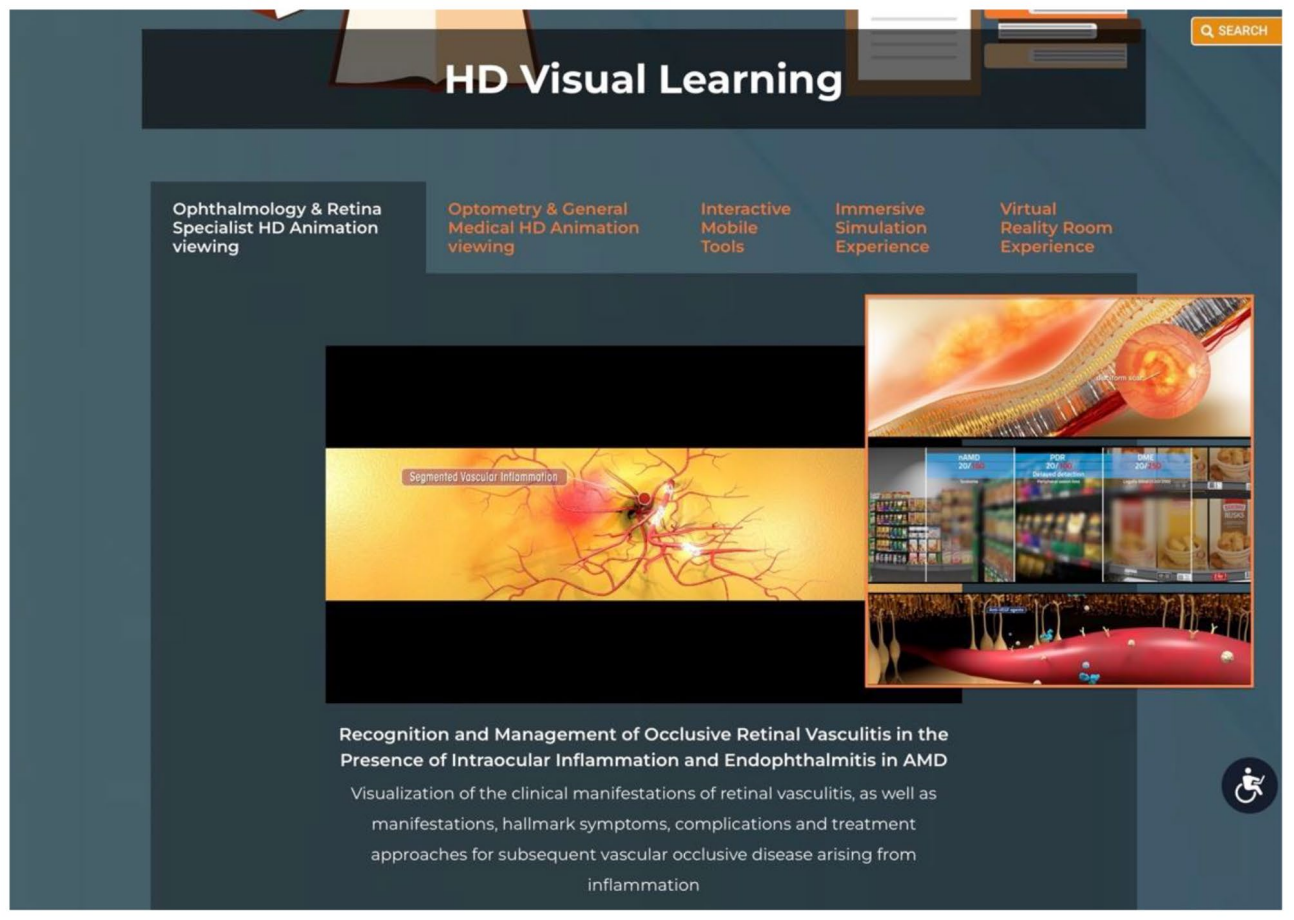
Virtual Reality Animations and Immersive Experiences to Increase Understanding of Disease Pathology and Treatment Approaches Images provided with permission by MLG, creators and curators of all VISION RELIEF content

**Fig. 4:**
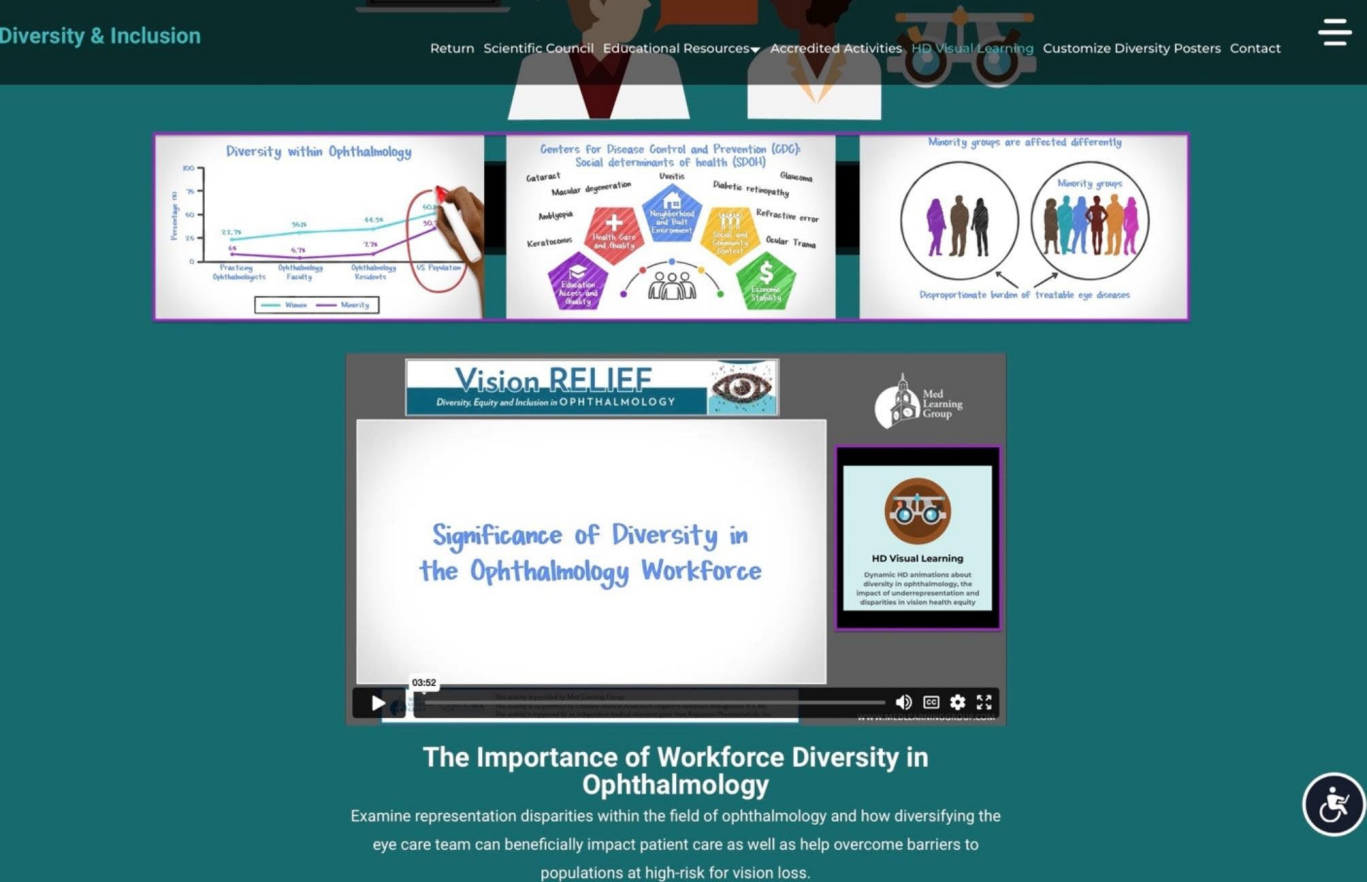
Video-Based Lectures to Help Reduce Health Disparities in Retinal Diseases in a Whiteboard Platform Images provided with permission by MLG, creators and curators of all VISION RELIEF content

VISION RELIEF is a continually evolving, modular educational platform that allows learners to engage with didactic content in conventional and illustrative modalities. Its therapeutic focus is on DR, AMD, RVO, and the appropriate application of anti-VEGF agents for these retinal diseases. The modular platform includes a virtual reality room/augmented reality learning lab; 33 live or virtually live learning programs; 38 learning tools for healthcare providers and patients; and enduring online programs. VISION RELIEF leverages cutting-edge technology (e.g., a Jeopardy-style quiz game to test knowledge, interactive clinical and patient experience simulations) to provide immersive content and features distribution partnerships with multiple respected medical societies, academic-research healthcare facilities, and federal agencies (e.g., National Institutes of Health, American Diabetes Association, American Academy of Ophthalmology, Johns Hopkins School of Medicine, Boston University’s Boston Medical Center), lending the platform credibility with audiences. Examples of learning programs and tools include expert presentations highlighting clinical trial data and guideline-based intervention with case-based discussion; a clinical trials dashboard that summarizes the latest research; whiteboard-style and virtual reality animations; interactive case simulations focusing on diagnostic ability and therapeutic determination; and virtual reality experiences. Figures 1, 2, 3 and 4 show examples of VISION RELIEF activities and content.

The pre-/posttest is conducted as follows: Prior to viewing the content, learners answer 3–5 questions that include both knowledge-based questions as well as 1–2 case study questions. Depending on the length of the activity, the time interval to complete is typically 30–60 min. Immediately after completing the program, learners complete a posttest with the same questions as on the pretest as well as an evaluation to assess satisfaction and intended practice changes. Scoring is based on the percentage of learners who answer questions correctly, with each question holding equal value. To receive AMA PRA Category 1 Credit for the program, learners must complete these assessments. Posttest results are only generated for participants who complete the program. Questions are only accessible through the registered CME activity; the website hosts connectivity links, providing a pass-through to the secure educational activity page. Thus, the results presented here are only from learners who completed both pre- and posttests.

The VISION RELIEF program provides non-bias education on both current and emerging therapies for DR/DME, AMD, and RVO. Content is peer reviewed to ensure there is no bias towards or against any therapies. The education is largely focused on VEGF-A inhibitors because of their support in clinical practice guidelines. The education is based on class of therapy as a whole, not individual drugs. All content is peer reviewed by a third party (i.e., healthcare providers) to ensure lack of bias and that the education is valid and scientifically sound.

## Results

### Participant characteristics

Two populations were studied for these analyses. For the pre/post-test knowledge/competency change analysis, scores from the full population of 10,786 learners were analyzed. For the practice change analysis with matched controls, scores from 7,827 learners comprised only of retinal specialists and ophthalmologists (i.e., likely providers of anti-VEGF injections) were analyzed.

As shown in Table 1, the full sample (N = 10,786) included the following areas of practice: retina specialists (12%), ophthalmology (20%), optometry (21%), primary care (19%), diabetes educator (2%), pharmacist/managed care (11%), and other healthcare provider (e.g., registered nurse, nurse practitioner, physician assistant; 15%). Among the matched analysis population (N = 7,827), 12% were retina specialists and 88% were ophthalmologists. A majority of the full sample was male (66% vs. 34% female) and White (66%). Geographically, most learners worked in the Northeast or Southeast.

**Table 1:** Baseline Demographics of the Full Sample

	Full Sample (N = 10,786)
Gender	
Male	66%
Female	34%
Race/Ethnicity	
White	66%
Asian	13%
Black/African American	8%
Hispanic, Latino, or Spanish	8%
Other	5%
Geographic Location	
Northeast	35%
Southeast	31%
Southwest	18%
Northwest	16%
Mean Number of Years Worked in Profession (SD)	9 (2.11)

### Change in knowledge/competence

As shown in Table 2, learners from all disciplines (N = 10,786) significantly improved their scores on all 5 items relating to early identification and treatment, identifying patients who could benefit from anti-VEGF agents, using guideline-recommended care in treatment decisions, recognizing the importance of screening and referral, and recognizing the importance of early detection and multidisciplinary care for DR (all *P*-values ranged from 0.003 to 0.004). When looking by subspeciality, learners in the “ophthalmologists and retinal specialists only” category (N = 3,285; Table 3) and those in the “optometrists, primary care providers, and diabetes educators only” category (N = 7,501; Table 4) also significantly improved their knowledge/competence on all 5 items (all *P*-values ranged from 0.001 to 0.004). Among all learners, the largest improvement in scores was observed for the item relating to appropriately identifying patients who could benefit from anti-VEGF agents (31% competence gain, *P =* 0.004). This item also represented the largest competence gain when looking at both the ophthalmologists and retinal specialists subgroup (28% gain, *P =* 0.004) and optometrists, primary care providers, and diabetes educators subgroup (33% gain, *P =* 0.004).

**Table 2:** Pre-Test and Post-Test Scores of Knowledge/Competence Among All Learners (N = 10,786)

	Pre-Test Score, M (SD) (% correct)	Post-Test Score, M (SD) (% correct)	Score Change, M (SD) (% change)	*P*-Value Score Change
Understanding of the importance of early identification and timely treatment of retinal diseases	M = 75 (SD = 6.01) (75%)	M = 92 (SD = 5.39) (92%)	M = + 23 (SD = 5.03) (+ 23%)	*P =* 0.003
Ability to appropriately identify patients who would benefit from anti-VEGF agents	M = 65 (SD = 9.41) (65%)	M = 85 (SD = 7.43) (85%)	M = + 31 (SD = 6.27) (+ 31%)	*P =* 0.004
Ability to utilize guideline recommendations/incorporating guideline-based practice into their treatment decisions	M = 64 (SD = 8.74) (64%)	M = 83 (SD = 6.94) (83%)	M = + 30 (SD = 5.11) (+ 30%)	*P =* 0.003
Recognition of the importance of screening and referral	M = 72 (SD = 5.58) (72%)	M = 88 (SD = 4.39) (88%)	M = + 22 (SD = 4.18) (+ 22%)	*P =* 0.003
Ability to recognize the importance of early detection and referral of diabetic eye disease, potential visual benefit from early initiation of DR treatment, and value of multidisciplinary management	M = 68 (SD = 7.83) (68%)	M = 86 (SD = 8.23) (86%)	M = + 26 (SD = 4.64) (+ 26%)	*P =* 0.003

Note: Mean score is out of 100 points possible

**Table 3:** Pre-Test and Post-Test Scores of Knowledge/Competence Among Ophthalmologists and Retinal Specialists (N = 3,285)

	Pre-Test Score, M (SD) (% correct)	Post-Test Score, M (SD) (% correct)	Score Change, M (SD) (% change)	*P*-Value Score Change
Understanding of the importance of early identification and timely treatment of retinal diseases	M = 83 (SD = 4.52) (83%)	M = 95 (SD = 5.10) (95%)	M = + 14 (SD = 4.71) (+ 14%)	*P =* 0.002
Ability to appropriately identify patients who would benefit from anti-VEGF agents	M = 76 (SD = 7.28) (76%)	M = 97 (SD = 6.82) (97%)	M = + 28 (SD = 5.56) (+ 28%)	*P =* 0.004
Ability to utilize guideline recommendations/incorporating guideline-based practice into their treatment decisions	M = 75 (SD = 9.32) (75%)	M = 93 (SD = 7.03) (93%)	M = + 24 (SD = 5.28) (+ 24%)	*P =* 0.003
Recognition of the importance of screening and referral	M = 86 (SD = 4.44) (86%)	M = 98 (SD = 2.28) (98%)	M = + 14 (SD = 4.07) (+ 14%)	*P =* 0.002
Ability to recognize the importance of early detection and referral of diabetic eye disease, potential visual benefit from early initiation of DR treatment, and value of multidisciplinary management	M = 82 (SD = 5.93) (82%)	M = 97 (SD = 5.55) (97%)	M = + 18 (SD = 3.71) (+ 18%)	*P =* 0.001

Note: Mean score is out of 100 points possible

**Table 4:** Pre-Test and Post-Test Scores of Knowledge/Competence Among Optometrists, Primary Care Providers, and Diabetes Educators (N = 7,501)

	Pre-Test Score, M (SD) (% correct)	Post-Test Score, M (SD) (% correct)	Score Change, M (SD) (% change)	*P*-Value Score Change
Understanding of the importance of early identification and timely treatment of retinal diseases	M = 71 (SD = 8.14) (71%)	M = 90 (SD = 5.71) (90%)	M = + 27 (SD = 5.62) (+ 27%)	*P =* 0.003
Ability to appropriately identify patients who would benefit from anti-VEGF agents	M = 60 (SD = 10.042) (60%)	M = 80 (SD = 7.69) (80%)	M = + 33 (SD = 7.26) (+ 33%)	*P =* 0.004
Ability to utilize guideline recommendations/incorporating guideline-based practice into their treatment decisions	M = 59 (SD = 8.41) (59%)	M = 78 (SD = 6.63) (78%)	M = + 32 (SD = 4.98) (32%)	*P =* 0.003
Recognition of the importance of screening and referral	M = 66 (SD = 5.86) (66%)	M = 84 (SD = 4.86) (84%)	M = + 27 (SD = 4.41) (+ 27%)	*P =* 0.003
Ability to recognize the importance of early detection and referral of diabetic eye disease, potential visual benefit from early initiation of DR treatment, and value of multidisciplinary management	M = 62 (SD = 8.49) (62%)	M = 81 (SD = 8.80) (81%)	M = + 31 (SD = 5.02) (+ 31%)	*P =* 0.004

Note: Mean score is out of 100 points possible

### Change in practice behavior

**Fig. 5:**
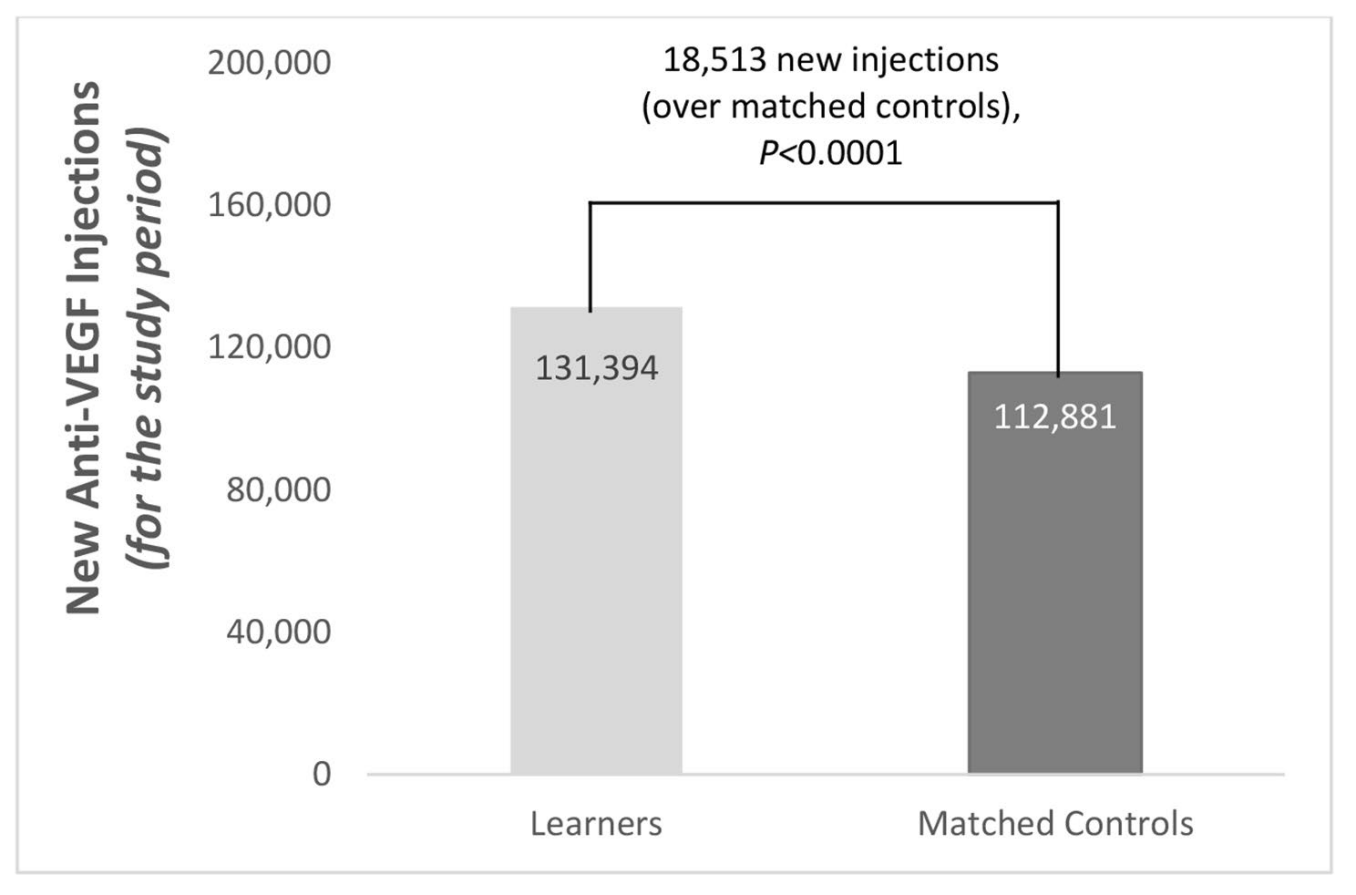
Anti-VEGF New Injections Among Learners and Matched Controls (N = 7,827)

The population of learners matched to a control of non-learners for the medical claims analysis (N = 7,827, 12% retina specialists and 88% ophthalmologists) demonstrated an improvement in practice behavior in the form of greater utilization of guideline recommendations/incorporating guideline-based practice into their treatment decisions. Specifically, compared with non-learners, learners demonstrated a greater increase in the appropriate application of anti-VEGF agents in practice, with incremental total injections for anti-VEGF agents increasing significantly before and after the intervention. Among learners, there were 18,513 more (new) anti-VEGF injections prescribed, a 16% increase from non-learners (Fig. 5; *P* < 0.001). Refill ratio was calculated using the national refill ratio, which is total treatments divided by new treatments during the study period, based on the national data. Applying a refill ratio of 6.86 to the increase in new injections observed during the study period yielded incremental 126,995 total injections (901,362 total injections among learners vs. 774,367 total injections among non-learners). The greatest change was among learners who were previously not using injections and were low-to-medium treaters. Specifically, when divided into decile ranks, those in decile 0 (N = 5,397) had 3,729 incremental new anti-VEGF injections, whereas those in deciles 1–3 (N = 1,815) had 12,825 incremental new injections.

## Discussion

This study is novel in several important ways. First, although previous research suggests CE programming can help improve clinician knowledge, skills, and behaviors, the literature on the practice impact of CE initiatives in retinal diseases and ophthalmology specifically is scant [29]. This study provides needed insight into the effectiveness of multicomponent, interactive learning on knowledge and competence outcomes in these disease states. Additionally, to our knowledge, this is the first study to link healthcare claims data with CE learning in retinal diseases and ophthalmology for the purpose of demonstrating how knowledge and competence gains can be translated into actual practice behaviors. Third, the scale of this study is sizable and includes multiple practice disciplines in this field, versus the small number of previously published CE studies in retinal diseases and ophthalmology, which tend to be conducted amongst professionals from a single discipline (e.g., optometry) [30].

This study of a modular, immersive CE initiative on retinal diseases found learners from multiple disciplines meaningfully enhanced their knowledge/competence about how to apply recommended interventions for the use of anti-VEGF therapies for retinal care. Specifically, all learners significantly improved their knowledge/competence scores relating to early identification and treatment, identifying patients who could benefit from anti-VEGF agents, using guideline-recommended care in treatment decisions, recognizing the importance of screening and referral, and recognizing the importance of early detection and care for DR. These patterns persisted when looking at knowledge/competence change by subspeciality categories. Moreover, ophthalmologist and retina specialist learners also improved their use of guideline-recommended agents (i.e., increased their number of anti-VEGF injections by 18,513 injections versus non-learners) post-education compared to a carefully matched control group, demonstrating that this CE platform was able to positively impact real-world clinical behaviors.

Although not focused on anti-VEGF therapy education specifically, 2 samples of learners from the platform from the 2022 American Diabetes Association (ADA) Conference and the American Diabetes Care and Education Specialists (ADCES) Conference similarly demonstrated improved knowledge/competence with exposure to the CE initiative. In the ADA sample, both live (n = 712) and virtual (n = 328) educational formats were analyzed. Of learners who completed pre-/post-testing (n = 644), there was a mean 32% competence gain (*P =* 0.005) in their ability to recognize the importance of early detection and referral of diabetic eye disease, the potential visual benefit from early initiation of DR treatment, and the value of multidisciplinary management [31]. In the ADCES sample, again, both live (n = 702) and virtual (n = 495) educational formats were analyzed. Of learners who completed pre-/post-testing (n = 493), there was a mean 32% competence gain (*P =* 0.004) in the ability to recognize the importance of early detection and referral of diabetic eye disease, the potential visual benefit from early initiation of DR treatment, and the value of multidisciplinary management [31]. Additionally, 99% of respondents (ADA n = 573, ADCES = 471) reported that the virtual reality program enhanced their learning experience, resulting in an increased likelihood for retinopathy screening referral (≥ 82%; ADA n = 558, ADCES n = 465) and greater willingness to engage in multidisciplinary care of DR patients (≥ 74%; ADA n = 527, ADCES n = 451).

Taken together, these findings suggest CE across a broad spectrum of eye and non-eye clinicians can help improve timely referral, screening, diagnosis, and treatment for retinal diseases. CE may also help address gaps in the healthcare system that serve as barriers to treatment (e.g., lack of clinician awareness about guideline recommendations for screening or referral by diabetes-related primary care clinicians and specialists). By educating both sides of the clinician care model (i.e., eye and non-eye specialists), CE could help provide more comprehensive and multidisciplinary care for patients with retinal diseases. However, it is important to note that CE can utilize a broad range of delivery formats (e.g., active vs. passive, print material versus video, animations, and 3-dimmensional imaging), which could vary their effectiveness at changing practice behaviors [29]. This suggests that not all education programs will be universally successful in terms of achieving professional and patient outcomes. The initiative studied here includes several features supported by the literature as being positively associated with practice change, such as the fact that it is multisensory, interactive, multifaceted, and self-directed [29, 32]. By contrast, more simplistic forms of CE, such as lectures or print materials, are akin to “spreading” information rather than helping students learn and retain information. This underscores why interactive content, content that allows learners to role play or practice implementing skills (as in virtual reality), and content that gives learners the opportunity to ask questions and receive feedback on performance has demonstrated better effectiveness than simple, noninteractive methods [33]. Similarly, a meta-analysis of the effectiveness of CE found active interventions (e.g., workshops, individual training) and mixed interventions (e.g., small group activities, role playing, feedback) demonstrated larger Pearson correlation effect sizes on improving physician knowledge or performance (both r’s = 0.33) than did passive interventions (e.g., lectures, print materials) (r = 0.20). Additionally, multifaceted education programs demonstrated large effects in improving physician knowledge (r = 0.69), as did case-based training (r = 0.64), whereas the effect size for conference and literature interventions was significantly smaller (r = 0.22) [34]. Finally, the use of virtual reality and 3-dimmensional technology in education platforms such as VISION RELIEF may help facilitate patient–provider and provider–provider conversations as well as better patient engagement in diagnosis and treatment planning, all of which may result in more collaborative care [35].

Increased uptake of anti-VEGF agents among eye care providers and educators is critical because anti-VEGF agents have demonstrated robust efficacy among certain eye diseases, such as DR [36], and are considered guideline-concordant care for AMD, DR/DME, and RVO [10–13]. Despite this established efficacy and support, anti-VEGF agents are underutilized in real-world settings compared with pivotal clinical trials, resulting in less vision gain for patients [19]. Improved clinician education about anti-VEGF therapies could help address differences in outcomes observed among real-world vs. clinical trial patients [19, 24]. The learning initiative studied here could help address this practice gap and demonstrates the merit of interactive, multifaceted CE while grounding development of future programming strategies in real-world evidence. For instance, although pre-/post-test results revealed that even retina specialists required more education on appropriate treatment choice in specific patient scenarios, the claims analysis established how CE improved quality of care via an evaluable change in adoption of appropriate guideline-based treatments. This intervention demonstrated the feasibility of achieving advanced outcomes across a far-reaching initiative and, going forward, could inform instructional design strategies and outcomes methodologies to facilitate such advanced results in other similar education platforms in other disease states.

### Limitations

Although the findings from this study are based on strong data from IQVIA, a good sample size, and carefully matched controls, some limitations should be noted. Our results on the knowledge/competence analysis in particular should be considered in light of disadvantages common to quasi-experimental designs such as pre/post-test designs, including the inability to infer causality and only determine associations; the use of participants who were not truly randomized and represent convenience/purposeful samples; and the fact that testing itself can introduce bias, such as sensitizing participants to the fact that their knowledge may be assessed following exposure to the intervention. For the practice behavior analysis, it is also important to consider that there is no way to control for participant exposure to additional educational information outside the intervention. It is possible learners attended other CE programs or engaged in independent study on the topic of anti-VEGF injections. This limits our ability to draw firm conclusions that the observed practice behavior change can be solely attributed to VISION RELIEF. However, this very large sample of learners who participated in this education program demonstrated significant changes in practice behavior that were not observed in individuals who did not participate in the program. It seems reasonable to extrapolate that the program had at least some influence on this observed discrepancy. Of note, less than one-third of the intervention participants completed pre- and posttests. In the authors’ experience, this is a very typical response rate for this type of education program and should not reflect poorly on the outcomes generated. Not every learner is taking the activity to receive credit. Learners receive a certificate if they complete the posttest and evaluation, but they can also view the content and not seek a certificate. Finally, without long-term data, it is hard to know whether knowledge “decay” or lack of retention of knowledge occurred in this sample, which could potentially influence future practice change and is a relevant outcome worth tracking in future studies.

## Conclusions

This analysis of an interactive, immersive, case-based, multidisciplinary medical education initiative focused on selected retinal diseases of AMD, DR/DME, and RVO yielded strong, positive outcomes in improved knowledge and competence, as well as real-world impact evidenced by robust practice behavior change as reflected in increased medical claims for anti-VEGF therapies, a guideline-recommended treatment. This study was novel in both its approach to linking CE learning in retinal diseases and ophthalmology to healthcare claims data, as well as in its scale. Collectively, the improvements reported here could have positive downstream effects on improving patient outcomes, although that would need to be determined with future follow-up studies looking at a broader range of study endpoints. This CE initiative was able to reach a large number of learners across a variety of specialties, closing the gap for impacting timely referral and diagnosis and treatment initiation, as well as incorporation of guideline-based therapies, which could lead to sizable impact in quality of care. Additionally, the ability to help clinicians enhance their use of guideline-recommended treatments, including anti-VEGF agents, could meaningfully impact real-world medical practice and result in patients achieving better vision gains than currently demonstrated outside the clinical trial setting. Furthermore, ongoing analysis of learners participating in 2022/2023 Vision RELIEF programming will provide longitudinal analysis of practice behavior impact in terms of treatment as well as new data on CE’s impact on referral and diagnosis rates.

## Data Availability

The datasets used and/or analyzed during the current study are available from the corresponding author on reasonable request.

## List of abbreviations

ADA: American Diabetes Association
ADCES: American Diabetes Care and Education Specialists
AMD: Age-related macular degeneration
anti-VEGF: Anti-vascular endothelial growth factor
BRVO: Branch retinal vein occlusion
CE: Continuing education
CRVO: Central retinal vein occlusion
DME: Diabetic macular edema
DR: Diabetic retinopathy
MLG: Med Learning Group
nAMD: Neovascular age-related macular degeneration
QOL: Quality of life
SD: Standard deviation
US: United States
